# Supplementary material for: Reliability of Static and Dynamic Network Metrics in the Resting-State: A MEG-Beamformed Connectivity Analysis
Source: Front Neurosci. 2018 Aug 3;12:506. doi: 10.3389/fnins.2018.00506 (PMC6088195; doi:10.3389/fnins.2018.00506)
Supplement: Supplementary file 1 [file Data_Sheet_1.docx]

**Supplementary Material**

**Reliability of Static and Dynamic Network Metrics in the Resting-State: A MEG-beamformed Connectivity Analysis**

S. I. Dimitriadis^1-6*^, B. Routley^1,4^, D.Linden^1,3-6^, K.D. Singh^1,4^

^1^Cardiff University Brain Research Imaging Centre, School of Psychology, Cardiff University, Cardiff, United Kingdom

^2^Neuroinformatics Group, Cardiff University Brain Research Imaging Centre, School of Psychology, Cardiff University, Cardiff, United Kingdom

^3^Division of Psychological Medicine and Clinical Neurosciences, School of Medicine, Cardiff University, Cardiff, United Kingdom

^4^School of Psychology, Cardiff University, Cardiff, United Kingdom

^5^Neuroscience and Mental Health Research Institute, Cardiff University, Cardiff, United Kingdom

^6^MRC Centre for Neuropsychiatric Genetics and Genomics, School of Medicine, Cardiff University, Cardiff, United Kingdom

**Section 1. External Dataset**

**2.1 Subjects**

15 healthy subjects (age $25.6\pm4.32$years, 8 women and 7 men) underwent two resting-state MEG sessions (eyes open) with a 1-week test-retest interval. For each participant, scans were scheduled at the same day of the week and same time of the day. The duration of MEG resting-state was 5 mins for every participant. The study was approved by the Ethics Committee of the School of Psychology at Cardiff University, and participants provided informed and written consent.

The preprocessing steps and the computations of static and dynamic functional connectivity graphs of this second dataset are described in the main manuscript. The parameters of the width of the temporal window and the stepping criterion were adapted from the first dataset.

**Section 2. Results**

S1-2 demonstrates the reliability of TR and OT for iPLV in analogy to Fig.9 and 10. S3-4 demonstrates the reliability of TR and OT for CorrEnv in analogy to Fig.12 and 13. The reliability of chronnectomics (TR,OT) for iPLV was high while for CorEnv the reliability of the TR reaches high acceptable levels. OT for CorEnv were lower compared to iPLV but higher than the original cohort. The results on this external validation dataset further support our results on the main cohort.


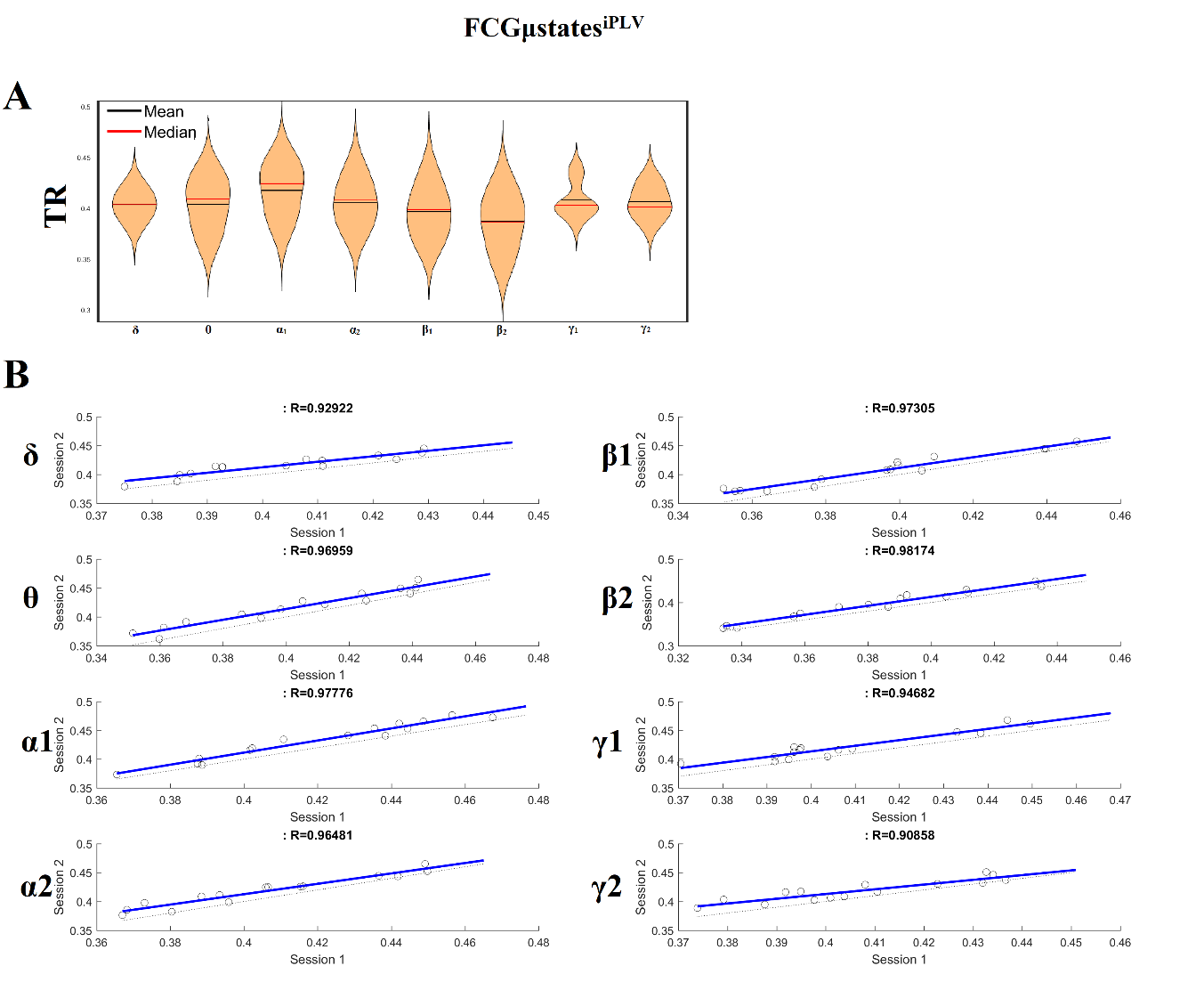


**S.1 Reliability of Transition Rates (TR) based on FCμstates^iPLV^ across frequency bands.**

1. Mean and median values of TR across subjects and scan sessions for each frequency band
2. Scatter-plot of subject-specific TR for both sessions with the corresponding fitted line for each frequency band. All the correlations were Corr.> 0.9 (p < 10^-7^)

Each blue circle corresponds to a participant.


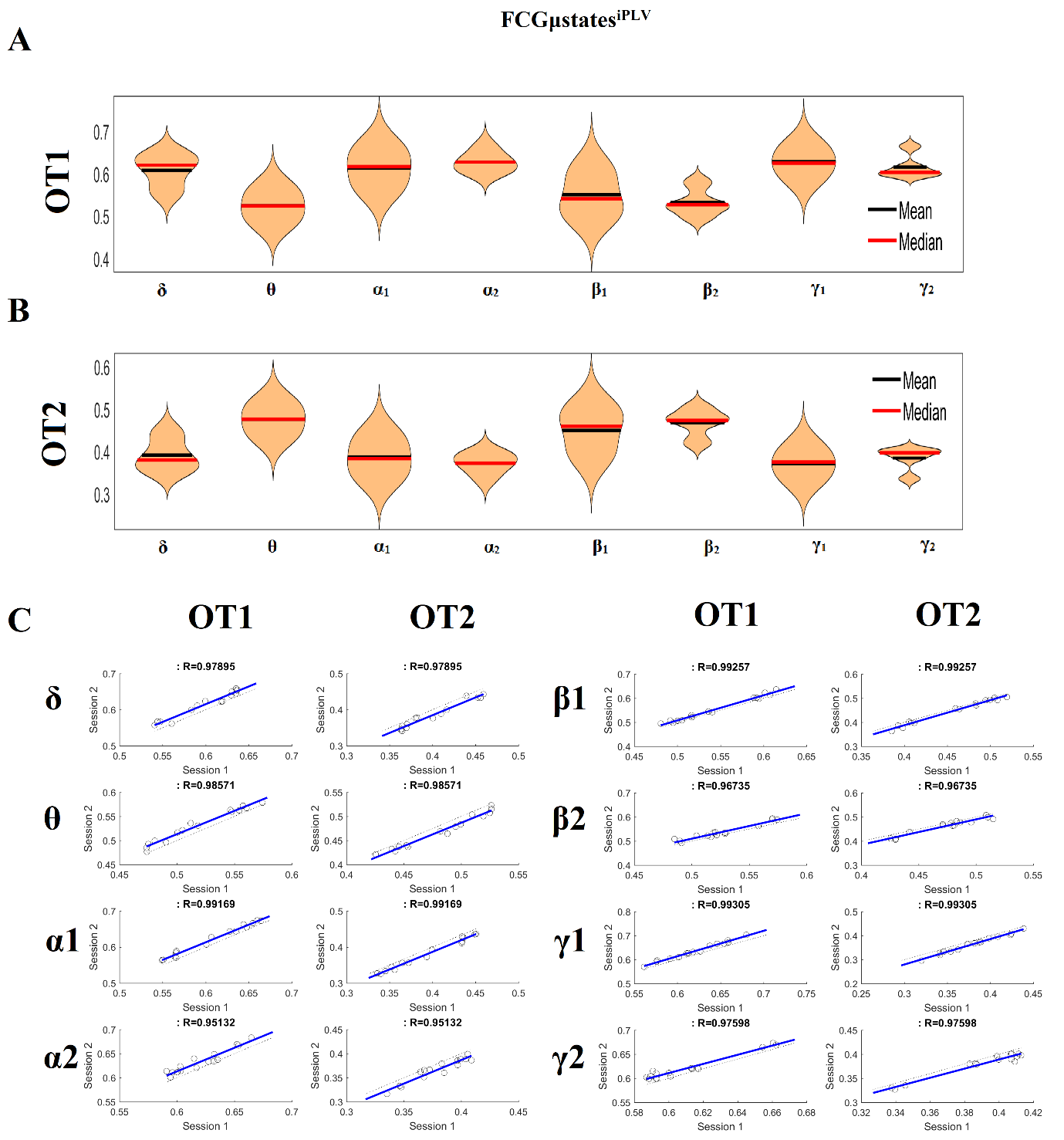


**S.2 Reliability of Occupancy Time (OT) based on on FCμstates^iPLV^ across frequency bands.**

1. Mean and median values of OC across subjects and scan sessions for **FCμstates^1^** and for each frequency band
2. Mean and median values of OC across subjects and scan sessions for **FCμstates^2^** and for each frequency band
3. Scatter-plot of subject-specific OC for both sessions with the corresponding fitted line for each frequency band. All the correlations were Corr.> 0.9 (p < 10^-7^).

Each blue circle corresponds to a participant.


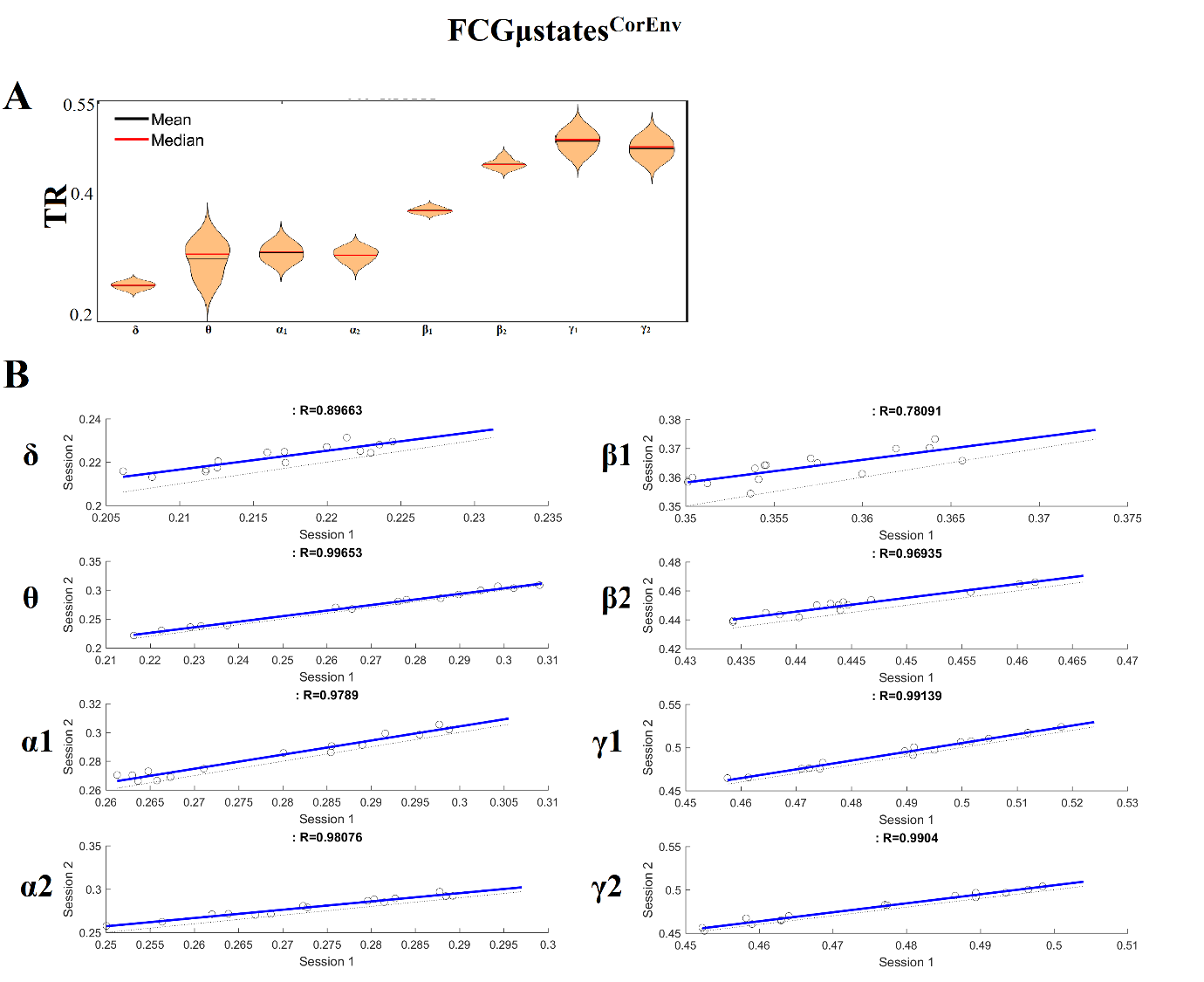


**S.3 Reliability of Transition Rates (TR) based on FCGμstates^CorEnv^ across frequency bands.**

1. Mean and median values of TR across subjects and scan sessions for each frequency band
2. Scatter-plot of subject-specific TR for both sessions with the corresponding fitted line for each frequency band. All the correlations were with the exception of β_1_ were high reliable ( > 0.9, p < 10^-4^).
3. Each blue circle corresponds to a participant.


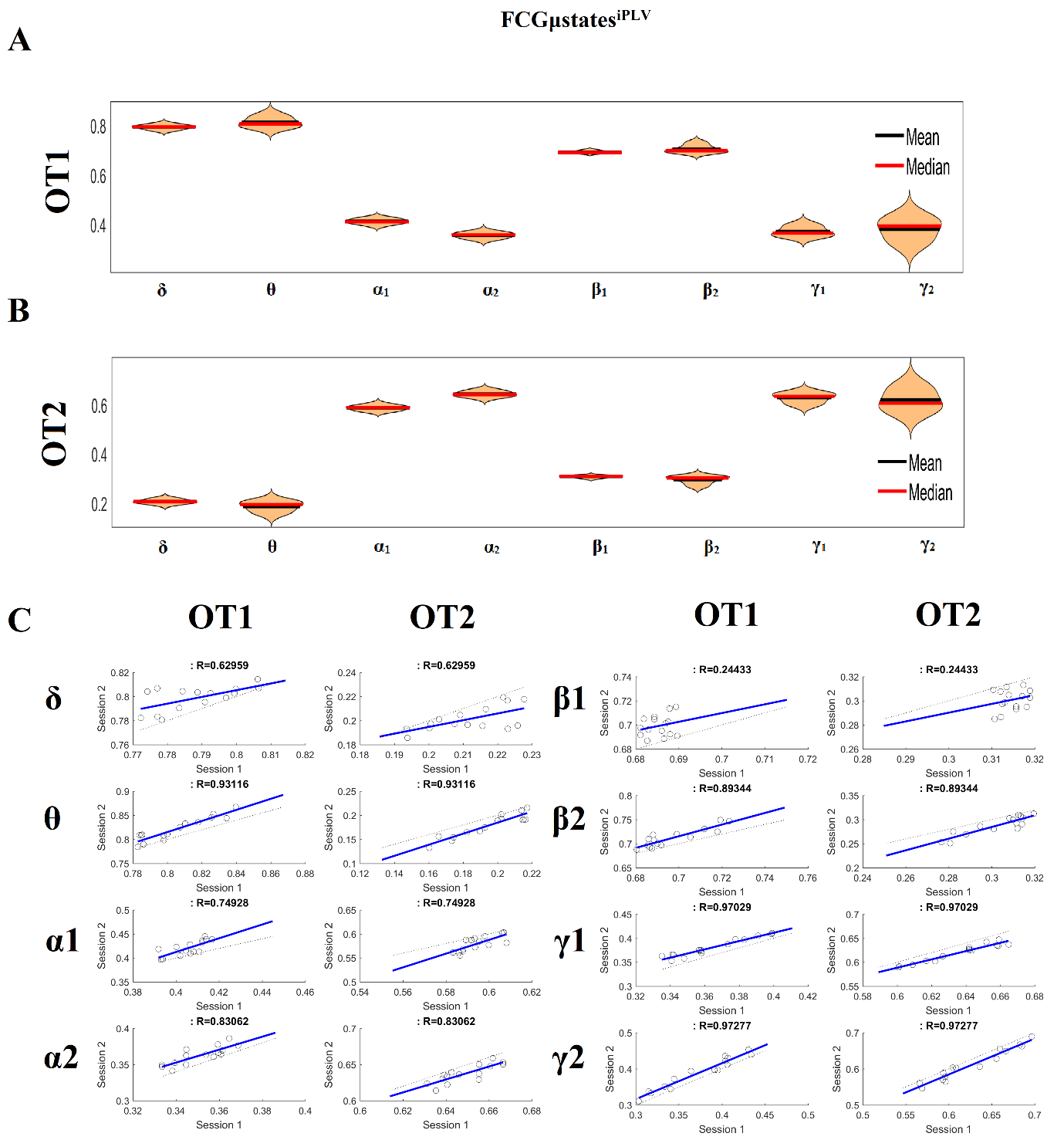


**S4. Reliability of Occupancy Time (OT) based on on FCGμstates^CorEnv^ across frequency bands.**

1. Mean and median values of OT across subjects and scan sessions for **FCμstates^1^** and for each frequency band
2. Mean and median values of OT across subjects and scan sessions for **FCμstates^2^** and for each frequency band
3. Scatter-plot of subject-specific OT for both sessions with the corresponding fitted line for each frequency band. Correlation values were lower compared to iPLV connectivity estimator (S3) Each blue circle corresponds to a participant.

**Section 3. AAL mapping to the Brain Networks**

Below, we give the mapping from AAL-90 to the five brain networks. The integers refer to the ordering of the AAL ROIs where one can find in the following link.

<http://neuro.imm.dtu.dk/wiki/Automated_Anatomical_Labeling>

**DMN: [5 6 9 10 23 24 25 26 31 32 33 35 37 38 39 40 47 55 69 85 86 88 89]**

**FP: [3 7 8 14 50 59 60 61 62 66]**

**O: [43 44 45 46 48 49 51 52 53 54 67 68]**

**CO:[** **11 12 13 15 16 21 22 27 28 29 30 34 36 41 42 56 65 71 72 73 74 75 76 77 82 83 84 87 90]**

**SM:[** **1 2 4 17 18 19 20 57 58 63 64 70 78 79 80 81]**
